# How Has Living with Intimate Partner Violence Affected the Work Situation? A Qualitative Study among Abused Women in Norway

**DOI:** 10.1007/s10896-016-9806-2

**Published:** 2016-02-24

**Authors:** Kjersti Alsaker, Bente E. Moen, Valborg Baste, Tone Morken

**Affiliations:** Department of Social Education and Social Work, Faculty of Health and Social Sciences, Bergen University College, Møllendalsveien 6-8, Postboks 7030, 5020 Bergen, Norway; Centre for International Health, Department of Global Public Health and Primary Care, University of Bergen, Bergen, Norway; Uni Research Health, Bergen, Norway; National Centre for Emergency Primary Health Care, Uni Research Health, Bergen, Norway

**Keywords:** Abused women, Intimate partner violence, Employment, Fear, Shame, Guilt, Recovery, Survivors

## Abstract

A qualitative study was conducted among 18 abused women from different parts of Norway to explore what paid work means for women exposed to partner violence and how living with an abusive partner affected their working life. Based on systematic text condensation analyses of their experiences as described in individual and focus group interviews, the study’s findings reveal two major themes. The first is about recovery and survival, and the other about the spillover of problems caused by a violent partner into paid work. Work was important to the women, as it represented time off from violence, contact with others who cared for them, and maintenance of self-esteem and self-confidence. Having their own money provided security and strengthened the belief that they could manage on their own. The spillover of intimate partner violence problems appeared through feelings of fear, shame and guilt at work.

## Introduction

The employment rate among women seeking refuge at women’s shelters in Norway is about 40 % (Alsaker et al. 2009; Vista Analyse 2012), while the general employment rate among women in Norway is about 75 %. The connection between abuse of women and employment is still very much a hidden issue in Norway as few researches have focused on this and as partner issues are perceived belonging to the private and work to the public area. It is therefore important to shed light on this issue. The aims of this study are to gain a better understanding of what paid work means for women in Norway exposed to IPV and how living with an abusive partner affected these women’s working lives.

### Definition of “Intimate Partner Violence”

The World Health Organization (WHO) defines Intimate Partner Violence (IPV) as “any behaviour within an intimate partnership that causes physical, psychological or sexual harm to those in the relationship” (Krug et al. 2002, p. 89). Such behaviour includes physical aggression, psychological abuse, forced intercourse and various controlling behaviours. Economic abuse is one type of controlling behaviour in a relationship. “Economic abuse involves behaviors that control a woman’s ability to acquire, use and maintain economic resources, thus threatening her economic security and potential for self-sufficiency” (Adams et al. 2008, p. 564). IPV may also be defined as “any act against another person that inflicts injuries or pain, instils fear or offense, and in this way makes the person to do something against her will, or to discontinue doing something that is in accordance with her will” (Isdal 2000, p. 36). The focus of the last definition is on reduction of autonomy. In general, employment increases autonomy and bolsters self-esteem when employees are in an environment of positive feedback (Waddell and Burton 2006). The quality of life among women seeking refuge from partner violence is very low compared to women who are not abused (Alsaker et al. 2006), and work may be important in enabling them to break out of violent relationships (Anderson and Saunders 2003).

### Effects of Intimate Partner Violence on Work

Research from the US has shown that women who have experienced intimate partner violence (IPV) find it difficult to stay in gainful employment due to problems at home (Rothman et al. 2007, Tolman and Wang 2005). Fear affects women’s employment through concentration problems at work and absenteeism from work (Samuel et al. 2011; Swanberg et al. 2007; Swanberg et al. 2006). Among the 120 IPV survivors in a US study, 78 % reported employment sabotage (Postmus et al. 2012). More than 60 % of the women in IPV relations in the US had to call in sick due to IPV (Swanberg et al. 2006). A study from Sweden found that women who had experienced IPV were absent from work twice as often as women who had not experienced IPV (Hensing and Alexanderson 2000).

### Policy Context of Intimate Partner Violence and Paid Work

Tradition, culture, religion and welfare in general may affect women’s opportunities to have paid work outside their homes. This makes it difficult to transfer knowledge about living in an abusive relationship and paid work directly from one continent to another.

In Norway, the women’s shelter movement has been strong for decades (Jonassen 2013), and new laws as well as political focus and decisions have made women better able to leave their abusive partners. Kindergarten for children from one to five years, for example, makes it easier for women to combine being a mother and having a paid job. Moreover, women in Norway have the same rights to education and paid jobs as men. Nevertheless, partner violence happens in intimate relationships at home, usually hidden behind doors, and many women still feel trapped in these relationships (Schei 1990). The lifetime prevalence of acts of IPV reported in a Norwegian population study was 27 % for women, and 8.8 % of the women reported having experienced life-threatening IPV (Neroien and Schei 2008). The socio-economic cost of intimate violence was estimated to be 4.5–6 billion NOK in 2010 (0.75–1.0 billion USD). The costs are based on an estimate to the effect that 75.000 to 150.000 people are exposed to intimate violence every year. Based on the number of people who seek help from the police and refuge at the Municipal Crisis Centre Services every year, about 8000 of these are in the group of people experiencing severe intimate partner violence. The main financial cost for the Norwegian society is considered to be loss of income, as many women exposed to IPV are either employed part-time or unemployed, or they may be out of work due to lasting loss of capacity to work (Vista Analyse 2012).

Global studies have found that intimate partner violence (IPV) against women is most common where gender roles are rigidly defined and enforced (Garcia-Moreno et al. 2006; Heise et al. 2002). Norway is a country with high levels of welfare, and it is classified as one of the countries where the gender gap is nearly closed (Hausmann et al. 2013). In this study, we explore what having paid work means to abuse women in a country with a high degree of gender equality. Further, we want to gain a better understanding of what paid work means for women in Norway exposed to IPV and how living with an abusive partner affected these women’s working lives.

## Method

### Sample

To find participants who had experienced IPV, we contacted women’s shelters and opened up for recruitment through the “snowball” method. Six women’s shelters located in various parts of Norway, both in rural and in urban areas, responded positively to the request to participate in this study. This was valuable, since opportunities for obtaining paid work vary with where one lives in the country.

The inclusion criteria for participants in this study were women who had experienced IPV, had paid work experience, and could speak Norwegian or English. All participants had left their partners. The one participant recruited by the “snowball” method had never been at a women’s shelter. All the other 17 participants in this study were women who had recently been residing at women’s shelters. The service scope of women’s shelters in Norway is to ensure the provision of a good, comprehensive shelter’s services to persons who have been subjected to domestic violence or threats of such violence. Individuals who need counselling or safe, temporary accommodation may contact the shelters without a referral or an appointment. Women seeking help at a shelter may themselves decide if they wish to stay for a night or two, or for a longer period. Shelters work on the principle of help to self-help.

The average age of the participants was 40 years, ranged from 24 to 53 years, the average length of employment was 11 years, ranged from 2 to 25 years, and the median time since separation from the partner was one year. All except one of the participants had children; on average they had two. Three of the 18 participants were from countries outside Europe; the remaining 15 participants were Norwegian. Most of the participants (15) were in paid employment at the time of the interview. They were in a variety of occupations, ranging from hotel worker to researcher, bakery worker, teacher, flight attendant, driver, nurse, social educator, retailer and consultant.

### Data Collection

The shelter’s staff invited informants to participate in an individual interview or in a focus group interview. The one participant recruited by the “snowball” method was asked by a friend who was living at a women’s shelter and had participated in this study. Nine individual interviews and three focus group interviews with two, three and four new participants, respectively, were conducted.

The interviews were recorded on audio files with the participants’ permission and subsequently transcribed. Additional written notes were taken during and just after the interviews. The individual interviews were unstructured interviews in which the role of the researcher was to listen and ensure that the focus remained broadly on the theme of *work*. The focus groups inspired the participants to make associations and share ideas, and opened up for discussion, revealing the complexity of the relationship between work and IPV. The individual interviews, however, provided more detailed accounts of the violence. In focus groups with more than two participants, the co-researcher was present as an observer. The other interviews were conducted by the first author alone. The interviews lasted 1–2 h. The data were collected in 2009.

All interviews opened with one main question, which was also given in the information letter about the study: “How has living in a difficult partner relationship affected your work situation?” This question was intended to allow for the unexpected and to provide an opportunity for following the informants’ own stories (Malterud 2012). It was, however, important to return to the main issue and repeat the question. The focus group interviews were based on some of the themes that emerged in the individual interviews, which were done first. The interviews covered issues such as being denied the opportunity to work, dealing with jealousy, being disturbed by their partner at work, and being concerned about their children’s situation while at work. In addition, the participants were asked about what impact the home situation had on their formal employment, and about how they felt about themselves as workers.

### Ethical Considerations

The interviewer received written consent from the 18 women. The participants completed a general declaration of confidentiality regarding the content of the focus group interviews. The safety and anonymity of the informants were major concerns, in line with the WHO’s special ethical guidelines for studies on men’s violence against women (Sullivan and Cain 2004). The study was approved by the Regional Committee for Medical and Health Research Ethics. All interviews were conducted in rooms where the participants remained undisturbed. The responses were de-personalized, and the participants were informed about their right to withdraw without any negative consequences. Emphasis was placed on ensuring that the individual participants should feel strengthened, not weakened, after having completed the interview. Pseudonyms were used for the quotes to ensure anonymity.

### Analysis

The researchers’ prior understanding of the study topic was based on several years of qualitative and quantitative research among women who had applied for refuge at Norwegian women’s shelters, with particular focus on IPV’s impact on women’s physical and psychological health, quality of life and life opportunities.

The data in the consent study were analysed using systematic text condensation based on Giorgi (1985) and modified by Malterud (2012). The analysis followed four steps:

1. Total impression—from chaos to themes: The first author read all the written material while listening to the audio recording in order to gain an initial overall impression and to identify the central themes in the women’s stories. The other authors read some of the transcripts.
2. From themes to codes: All authors identified and organized meaning units.
3. Condensation—from code to meaning: Data were reduced to a decontextualized selection of meaning units sorted as thematic code groups across individual participants. All Authors discussed the meaning units and categorized them into thematic code groups.
4. Synthesising: Finally, we collaborated on the synthesis process, in which we moved from condensation to description and concept identification. During this process we returned to the main text data several times to find the quotations that would best describe the main findings. The recordings were transcribed by the first author and by a secretary.

### Discussion of Methods

All except of one of the women in the current study had been in a women’s shelter, so the results may primarily be applicable to abused women who contact women’s shelters. All women who participated had left their abusive partner. This characteristic shows that they are selected, and they can be described as belonging to a “ready to change group” (Karakurt et al. 2014). This type of selection indicates that leaving the abusive partner is related to the ability of reflection upon paid work.

The geographic spread of the informants and their different occupations provide variation in the data. The combination of individual interviews and focus groups was found to increase the richness of the data collected. Focusing on experiences through the detailed stories ensured a rich data material and more depth to our understanding of the complexity of the phenomenon in question, IPV in relation to work. The grouped data facilitated the focus on work when the individual unstructured interviews gave a deeper insight in fear and the spillover of the violence to work. The individual interviews gain a better understanding of the value and problems linked to paid work for the abused women. However the concept “shame” was first used in a focus group and the discussion followed opened up for new insight.

## Results

Most of the participants had no problems talking about their experiences. However, some of them needed breaks in the interviews due to emotional reactions. Two main themes were identified: “Survival and recovery” and “Spillover of intimate partner violence into paid work”.

### Survival and Recovery

“Work provides income, freedom and self-esteem”

During reflection about work and partner-violence after having left their abusive partners, the women discovered how employment had been, and this still was very important to them. Being a skilled worker gave a sense of acceptance and worth, and helped build self-esteem, helped provide a sanctuary from violence, and allowed for a wider scope of action, as the following examples illustrate:

Susan: *But for me it has been so incredibly important to go to work*, *because out there in working life we are worth something. So maybe that is why I survived and didn*’*t throw myself off the balcony*, *because I experienced so much acceptance at work.*

Tine: *My job has always been my sanctuary. I have been able to be a professional there*, *you know*—*to concentrate on things.*

Farris: *For me it*’*s a kind of a freedom. It*’*s good for me to work*, *because then my head is somewhere else.*

Of particular importance was the freedom to think about work, the freedom from violence, and the fact that they had an independent income that facilitated a wider range of actions. Some of the participants said that their income had made it possible to take a taxi to the women’s shelter. All of the women described how having their own money provided security and strengthened the belief that they could manage on their own.

Kelly: *As long as I wasn*’*t working*, *I didn*’*t have my own account number and my own account*, *so I could never even get a taxi*, *no matter how much I wanted to get to the women*’*s shelter.*

Carol: *The most important thing*—*I knew that if I was going to manage to get out of it then I had to have a safe base*—*your job is your livelihood*, *it*’*s what you pay with. It*’*s everything.*

As the quotes illustrate work was experienced as being of high importance to women living in abusive relationships since work represented a path to freedom and an opportunity to use their resources, in addition to providing a feeling of worth and a better life.

#### Spillover of Intimate Partner Violence into Paid Work

The women described spillover of intimate partner violence into paid work through feelings of fear, shame and guilt, as well as through their need to get help from colleagues to do their job satisfactorily. The feeling of fear of their partner affected their work situation in different ways. They talked about how they felt strained and were looking over their shoulders even at work. Being interrupted by their partners or ex-partners and remembering the threats of hurting and killing them, taking the children away from them or making their future miserable by destroying their reputation, made it difficult to concentrate. For some of the women, the partners’ threats had dominated their earlier lives and continued to do so even after they had left their partner, at work, as well as at home. They were afraid of being stalked on the way to and from work. Some described how fear caused concentration problems, which again was a source of new fears, such as fear of giving patients the wrong medication.

Gaby recounted the following: *I work with medication as a nurse*, *and I hand out drugs. To go to work is one thing*, *but then you have to concentrate and remember*, *and then suddenly you might get a call from one of your children saying*: ‘*Now I do not want to be here*, *I do not want to be with daddy*, *you must come home*, *mummy*;’ *and then it starts churning*, *but then you have to start concentrating*: *What kind of medicine was it*? *Yes*, *I*’*ll take that one with me*, *and it might be completely wrong*, *and that*’*s very dangerous. So I stopped working in that job as a home-based care provider.*

They were also afraid of failing to care for their children when leaving them alone with their partner while they were working shifts. For instance, they were worried that their partner would not pick up the children from kindergarten or school as agreed; or they were afraid that the partner would leave the children alone at home. Some asked their employers for day shifts only.

Pernilla described this as follows: *When our youngest was three months old and the oldest was four*, *he left them alone while I was at work and he was supposed to look after them*; *and then I thought*: *I cannot go to work*, *I cannot do anything.*

Some of the participants experienced stalking that interrupted their workday. Gina said: *I never answer the telephone at work. If any known customer asks for me* (*most of our customers are known*) *I will call them back. If someone else calls*, *my boss will tell them that I am not available. My ex-partner gets others to call me and stalk me. They say that they will rape and kill me. I have a secret telephone number for my own phone*, *but they still find it.*

Some talked about how they took a new road to and from work to avoid meeting the partner, and some described how colleagues reorganized their job to protect them, as described by Susan: *At work as a community care nurse my colleagues said they would help me. They drove me around in a big district*, *and he could not know my shift schedule or the address at which I worked.*

Two of the three foreign women feared being killed by relatives other than their partner because they had received threats from their mothers-in-law and other relatives after they left the perpetrator. One of them also feared that the children would be moved to their home country, where women are denied the right to contact the children without the father’s permission.

Four of the participants had to leave their jobs as a consequence of moving to another part of the country for safety. Two of them had new jobs and two hoped to get new jobs. Three other participants had received sickness certification by their physicians since they could not concentrate well enough to work; one participant was out of work and was living on her savings.

The impression gained from the women’s stories was that living with abusive partners was a cause of fear both when living with the perpetrator and after leaving him. Fear had an impact on the women’s ability to relax and concentrate at work. They felt strained and anxious at work, and their job performance did not reflect their abilities.

Shame and guilt as consequences of IPV-related lies emerged as significant features in the women’s work lives through the fact that they did not want to reveal their shameful secret of being abused by their partner. The concepts of “shame” and “guilt” were not introduced by the researcher during the interviews. The concept of “shame” implies feelings of being insignificant and worthless, both in one’s own eyes and in others’ eyes, in addition to a desire to hide or escape. Some of the participants spoke of such experiences in relation to their roles as employees without using the concepts of “shame” or “guilt” when describing their experiences.

By causing the women to arrive late or miss work, or by making them extremely tired by keeping them awake all night, the partners continued to cause problems for the women’s work situation in a manner that generated experiences of shame. Frequent telephone calls to check up on the women also caused difficulties at work.

Some of the participants revealed shame and guilt related to living in an abusive relationship and not breaking out*.* They expressed being unable to tell the truth about why they were late for or absent from work. They felt that they had to keep up appearances and hide their shameful secret. This gave rise to even stronger experiences of shame, as described in the following statement by Susan: *People have suspected something*, *but I have been really good at keeping up appearances*, *because that*’*s more important than anything*—*that no one finds out*, *because it*’*s a sign of failure that you are in a relationship like that and cannot get out of it.*”

The “sign of failure” was associated with a feeling of “shame”, which seemed to be related to not breaking out of the violent relationship. Sometimes the women told lies to avoid revealing their shameful secret. According to the women, it was not easy to tell someone that their partners failed to pick up the children at kindergarten for the fifth time or that they left two young children completely alone in the flat. Some had experienced problems with walking because of sexual abuse, and others had visible bruises because of physical violence—things that also contributed to the experience of shame. Lena said that she lied to avoid having to participate in social events or teambuilding events at work. She could not tell her colleagues the truth about her partner, who did not allow her to participate and would hurt her if she did.

Lena: *You have no idea how much I had to lie. I was always saying*: ‘*No*, *I don*’*t have a babysitter and he will be away at that time* …’ *It was just terrible. I am an incredibly honest person and very sceptical of people who lie*, *so to have to do it myself*, *and cover up... just terrible* … *And I*’*m a very sociable person*, *so I want to be together with the others.*

The women’s stories moreover described thinking about themselves as “stupid”. Many felt that they wore the wrong clothes, that they had to refrain from expressing their thoughts, or that it was difficult to talk to other people. Expressions such as “low self-esteem” and “low self-confidence” were used several times. Harriet and Doris described the feelings as follows:

Harriet: *Of course it affected me at work. I developed problems talking to people*, *I felt stupid. I do not know how to explain it*, *but I felt like I was a nobody.*

Doris: *My self-esteem is so low that you sort of don*’*t... Well*, *in the end you believe that it*’*s your fault and you are so deeply ashamed that you*—*a grown adult*—*are willing to live like that*, *in a way. As if you don*’*t deserve any better*; *and that*’*s not the truth*, *but you only realize that once you manage to get out of it*, *don*’*t you*?

The problems in the violent relationships affect the women’s work situations through negative feelings. Fear, shame and guilt are all experienced as negative feelings. The women’s stories showed that they experienced these feelings intensely in relation to their jobs.

## Discussion

In this study, women who had experienced abuse from partners and had left them, had discovered that employment had been still was very important to them. Having a paid job increased their self-esteem and the range of options they had with regard to acting independently and being able to have a better life.

The women in our study stressed that work was valuable for building them up, that it represented a sanctuary from violence, and that it provided a range of opportunities to act independently. Our findings are supported by other studies, which have revealed how employment helps women who experience IPV by improving their finances, promoting physical safety, increasing self-esteem and improving social connectedness (Cheng 2013; Logan and Walker 2009). The ways in which employers support women with regard to IPV’s impact at work may be significant in encouraging them to continue working in future. Evaluating oneself on the basis of positive reactions generates an experience of self-worth, of being valuable, whereas violence generates negative self-evaluations and feelings such as shame and guilt (Mead 1967; Wilson et al. 2006). Women exposed to intimate partner violence need positive feedback, and employment provides opportunities for such feedback. Shame and guilt are negative feelings about oneself and the opposite of self-worth.

Intimate partner violence creates fear that spilled over into the work situation. According to the women, fear forced them to act against their will in terms of missing work, starting late or leaving early, as well as making them tired and distracted. The women in this study described intense feelings of fear of being injured, as well as fear for their children when they were absent. Fear was also related to stalking by the partner or ex-partner, or proxy stalking. Fear spilled over and created concentration problems at work. Perpetrator intrusion at expected safe places such as work influenced their ability to concentrate, as found in the studies from the US (Logan and Walker 2009; Logan et al. 2007; Swanberg et al. 2007; Swanberg et al. 2006). Fear may be a part of post-traumatic stress disorder (PTSD) after leaving the perpetrator (Kimerling et al. 2009), but among our participants fear was also experienced in relation to actual threats.

The work-related problems caused feelings of shame and guilt, and these feeling increased when the women were not open about the reasons for those problems. Also, in Norway, IPV is still an under-reported public health problem and associated with taboos (Heise et al. 2002). Some of the participants said that the shame they experienced was related to the fact that they did not leave their partners, and to the fact that they had themselves “chosen” their bad partners as husbands. The social stigma of not being a successful marriage woman and not a proper worker, as well as the fact that being a single partner often is hard economically may be other reasons for not leaving the partner.

The finding of a significant spillover of shame and guilt into the women’s paid work was unexpected. In a country where gender equality is high and the fight against intimate partner violence is addressed through laws and reports, we would not expect the victims to express the experience of shame in so many ways. In a recent qualitative study from Taiwan, the participating women’s experiences of shame after terminating abusive relationship are understood in relation to breaking norms in a patriarchal society (Hou et al. 2013). Our study of shame reveals shame in relation to breaking cultural codes. The participants’ work-related problems, such as arriving late or leaving early from work, being tired, lying and being disturbed at work by one’s partner, thus creating conditions of stress, are very much unacceptable in a Norwegian work culture. Attitudes as “blaming the victim” and “keep your private problems private” are some Norwegian culture norms that can make it difficult for the abused women to telling anyone about their IPV problems. The dichotomy between the private and the public have been and are still central in the covering up domestic violence” (Holst 2002; Kymlicka 1990).

Shame and guilt due to IPV led the women to conceal the fact that they felt forced to act in ways not in accordance with the dominant cultural codes. The fact that they felt that they could not reveal the shameful secret that they were abused before they were in a process of leaving is supported by a recent study revealing that the attitudes of others in “blaming the victim” are reduced if the abused women try to leave the perpetrator (Halket et al. 2014). Such attitudes are still widespread in the EU countries at the start of the millennium, contributing to a climate of social acceptability of IPV against women (Gracia and Herrero 2006). Many of the examples of abuse told by the participants in this study are power and control strategies that keep women in the subordinate position to fit the age-old/conservative perceptions about manhood and womanhood. In an egalitarian society such examples may be even less acceptable than in other less egalitarian societies. As Norway is a country with high levels of welfare and gender equality (Hausmann et al. 2013), this may also strongly increase the feeling of shame associated with being abused.

Shame and low self-esteem are closely interconnected (Honneth 1995; Lawrence and Taft 2013). Feelings of shame imply feelings of being of less worth than others, of being excluded, or of being different in a negative way, such as feelings of being stupid. The deepest feeling of shame is defined as a strong feeling of not being worthy as a human being. In the literature the concept of “shame” is related to self-esteem, openness and guilt (Wyller 2001), and reflects a concern with a threatened self (Honneth 1995). The philosopher Løgstrup described how two paradigms are linked to shame such that: 1) shame that is felt because actions or attitudes are not in accordance with dominant cultural codes, and 2) shame that prevents one from crossing other people’s personal boundaries (Løgstrup 1984). Shame that prevents one from crossing other people’s personal boundaries is a positive form of shame that involves empathy, respect and sensitivity to other people’s boundaries. However, some people do not feel shame or guilt, even when they behave in ways that offend others and violate their boundaries. With regard to shame, Løgstrup uses the concept of “shamelessness” (Løgstrup 1984), which he maintains may induce shame in others (Martinsen 2009, p. 102). Persons who lack a sense of shame and guilt seem to have no limits or boundaries for how they can behave towards other people. We will indicate that women exposed to IPV are becoming bearers of their partner’s shamelessness by feeling shame on their behalf. Shame is, along with fear, described as one of human beings’ most strongly negative feelings (Løgstrup 1984).

Hooge et al. (2008) maintains that shame can also be a motivating feeling. When you feel ashamed of not doing your best, for example by not being properly prepared for an exam, you may be motivated to do better next time. Thus, work-related shame may potentially also motivate women to leave their abusive partners, as emphasized by Hooge et al. (2008). Honneth (1995) also maintains that shame may become a motivational impetus in a struggle for recognition. Thus, there is a need to link these feelings to the disrespect that prevails in IPV and to a social movement articulating that disrespect (Honneth 1995).

Shame has also been found to be associated with post-traumatic events related to loss of face, humiliation, shame in others’ eyes, and loss of roles (Wilson et al. 2006). In a Canadian study, the best predictors for unemployment were psychological violence and post-traumatic stress disorder (PTSD) (Kimerling et al. 2009). IPV has also been found to be a leading cause of PTSD among women in Spain (Pico-Alfonso 2005). According to Lawrence and Taft 2013, the relationship between shame and aggression, IPV and PTSD may be important and should be included in our understanding of IPV and traditional batter-prevention programmes.

Some of our participants reported that they felt a need to be extremely skilled in their jobs. This may be viewed as a reaction to the shame and guilt inflicted upon them through abuse (Honneth 1995; Niedenthal et al. 1994), as well as a means to support their feelings of self-worth. The breach of norms involved in telling lies, arriving late for work or not turning up at all may affect women’s experiences of their own identities (Mead 1967). Thus, being employed both strengthened and weakened their self-esteem as long as their work-related partner problems were kept hidden.

### Limitations

Norway may be different from many other countries insofar, as Norway has the highest rate of employment among women; it is also possible for women to continue working as single parents (Scott et al. 2010). However, the women in our study were still likely to report shame and guilt, and they had experienced serious partner violence. Since our participants were between 24 and 53 years of age, we do not know whether the findings are relevant for younger or older women. Moreover, we have no knowledge about men exposed to IPV. Three of the informants came from countries outside Europe. Their experiences of the impact of IPV on employment did not differ much from those of the ethnically Norwegian informants, except in relation to fear. Possible memory bias may exist, as the women’s experiences were mostly retrospective.

## Conclusion

Being employed was both positive and difficult for the abused women. Paid work both strengthened and weakened their self-esteem, as long as their work-related partner problems were kept hidden. Paid work represented survival and recovery through freedom from violence and contact with others who cared for them, and this was important for the maintenance of self-esteem and self-confidence. Moreover, having an income facilitated a wider range of opportunities and a wider scope for action. The women in this study reported that the intimate partner violence strongly affected them and manifested itself in the form of feelings of fear, shame and guilt at work. These feelings have to be included and addressed in our understanding of spillover of intimate partner violence into paid work among the survivors, their colleagues and their employers. The importance of having a paid job, as well as the need to address the spillover of negative emotions relating to violence, must be part of our understanding of and addressed in our research on intimate partner violence and paid work.

### Implications of the Findings on Research, Practice and Policy

This study reveals that shame, guilt, fear and low self-esteem made it difficult for women experiencing IPV to reveal their life situations and ask for help. More research is needed to establish the prevalence of these problems at workplaces in Norway and other countries. Careful longitudinal intervention studies are needed in order to develop the best methods for helping abused women handle these problems.

The women’s shelter movement and white papers (Ministry of Justice and Public Security NOU 2003, 2008, 2013) on IPV may be seen as a part of social movements articulating disrespect (Honneth 1995). The very low quality of life among abused women in Norway compared to women who are not abused (Alsaker et al. 2006) indicates that when human rights, laws and cultural codes are denied in the private sphere, the private becomes public and spills over into all aspects of life, including work life. The stories told by the women in this study may enhance our understanding of how this happens. When women living in a modern society with a high degree of gender equality and high level of welfare support tell such stories, they strikingly illustrate how destructive IPV is, and how IPV severely affected these women’s lives.

In this study the participants had received an information letter and agreed to participate, so they were prepared to talk about how the violence affected they work. This is rarely the situation in other settings (for instance when health personnel meet women with similar problems). Remembering can be hard, and it is important to understand that women with these types of experiences might be emotional in the interview situation and may need time to be able to talk about it. As a researcher—as well as in practice as a clinician—acceptance and respect for the zone of untouchability are important, as are being able to deal with the survivors stories of pain (Martinsen 2006).

Empowerment through talk may also enhance the survivors’ abilities to find their own way towards a fruitful life without fear and financial loss. Different forms of support from supervisors in the workplace were desired among abused women, and these forms reflected stages of behaviour change in an abusive relationship. When women were breaking away from the abusive partner they desired “support-in-every-way” (Perrin et al. 2011).

Employers and co-workers need to know that negative incidents at work may be a result of IPV. It may also be important that social and welfare services that contributes to the financial security of the victim, maintain a specific focus on helping abused women to keep their jobs or find new ones if they have to move to a different part of the country. Work may have a positive impact on women’s self-esteem and income, function as a social insurance and empower clients in IPV situations to be financially independent from the abusive partner. As a Taiwanese study (Hou, 2013) suggested, “reconstructing the self” may also be an essential issue for Norwegian abused women after terminating abusive relationships. Work and education may be important for empowerment and for the recovery process after leaving the perpetrator. In addition, employers and co-workers, doctors, psychologists and other practitioners, as well as the abused women themselves, need to know more about how to reduce the negative feelings of fear, shame and low self-esteem that make employment problematic.
